# THERAPEUTIC ALLIANCE AND RELAPSES OF SUBSTANCE USE

**DOI:** 10.1192/j.eurpsy.2023.1396

**Published:** 2023-07-19

**Authors:** N. El Moussaoui, B. Sofiya, T. Aicha, F. El Omari

**Affiliations:** 1 Psychiatry, Arrazi Hospital of Sale, Salé, Morocco

## Abstract

**Introduction:**

Therapeutic alliance is the key to ensure adequate and sustainable care in psychiatry.

The therapeutic alliance has been the subject of a great deal of psychotherapy research, and evidence from numerous empirical studies suggests that a strong patient-therapist relationship predicts favorable treatment outcomes and continues to be regarded as an important aspect of the therapeutic process

**Objectives:**

The purpose of this studie is to evaluate how the development of therapeutic alliance dimensions was associated with srelapses prevention.

**Methods:**

This is a prospective, observational, cross-sectional study, carried out in the department of addictology at Ar-razi hospital in Salé using a therapeutic alliance measurement scale, and evaluating relapses’ frequency and duration of abstinence. The therapeutic alliance (TA) score was measured using Working Alliance Inventory.

**Results:**

The results call upon the concepts of anomie and attachment, which seem to play an important role in the follow-up and prevention of relapses, indicating the need for a global approach to care and the involvement of health and social professionals, where empathy must find its place.

**Conclusions:**

Decades of psychotherapy research suggest that the strength of the relationship between patient and therapist is a common factor that is associated with treatment response. In the context of relapses specifically, most of the studies reviewed found evidence for a significant alliance-outcome relationship.

**Disclosure of Interest:**

None Declared

